# Supplementary material for: Content-rich biological network constructed by mining PubMed abstracts
Source: BMC Bioinformatics. 2004 Oct 8;5:147. doi: 10.1186/1471-2105-5-147 (PMC528731; doi:10.1186/1471-2105-5-147)
Supplement: Additional File 2 — The original results of the above study (non-essential files are deleted to keep the file size under the limit set by BMC bioinformatics). [file 1471-2105-5-147-S2.bz2 › chilibotAdditionalFile2/dip10/218ID9694899E726/html/IL6R_IL6.html]

 


 **IL6R** and **IL6** 
  
Found 377 abstracts in PubMed, retrieved 10.  
 

 What does Google say? 
 PDF only 
| .edu only 

---

**Interactive relationship** (e.g. stimulation, inhibition, etc)

**Stimulatory relationship**- The mRNAs of both receptors of IL 6  [ **IL6** ] , the soluble  **IL6R**  and gp130, were increased subsequently to IL 6  [ **IL6** ]  mRNA.  Ref: 12733076 Pflugers Arch< MedlineTA>Pflugers Arch,
**Neutral relationship**

**Non-interactive relationship** (e.g. studied together, co-existance, homology, etc.)

- An aminopeptidase, ARTS 1, is required for interleukin 6  [ **IL6** ]  receptor  [ **IL6R** ]  shedding.  Ref: 12748171 J Biol Chem< MedlineTA>J Biol Chem,
- Aim To measure serum concentrations of the soluble interleukin 2 receptor sIL2R, soluble interleukin 4 receptor sIL4R, soluble interleukin 6  [ **IL6** ]  receptor  [ **IL6R** ]  sIL6R, and soluble tumor necrosis factor alpha receptor I and soluble tumor necrosis factor alpha receptor II, during the perinatal and early neonatal period, in order to evaluate their role in activation of immune response in labor and the first days postpartum.  Ref: 12857603 Mediators Inflamm, 2003
- Using the recently engineered designer cytokine IC7, which induces an active heterodimer of the LIFR and gp130 after binding to the IL 6R, and several receptor chimeras of the LIFR and the interleukin 6  [ **IL6** ]  receptor  [ **IL6R** ]  IL 6R carrying the CBM of the IL 6R in place of the COOH terminal LIFR CBM, we could assign individual receptor subdomains to individual binding sites of the ligand.  Ref: 12731865 Biochemistry, 2003
- Cellular cholesterol depletion triggers shedding of the human interleukin 6  [ **IL6** ]  receptor  [ **IL6R** ]  by ADAM10 and ADAM17 TACE.  Ref: 12832423 J Biol Chem< MedlineTA>J Biol Chem, 2003
- Part II Significance of anti interleukin 6  [ **IL6** ]  and anti soluble interleukin 6  [ **IL6** ]  receptor alpha  [ **IL6R** ]  antibodies in a standardized murine contact burn model.  Ref: 12771624 Crit Care Med, 2003
- 2 mediators and receptors of immunity, i.e. neopterin, soluble interleukin 2 receptor sIL 2R, soluble interleukin 6  [ **IL6** ]  receptor  [ **IL6R** ]  sIL 6R, soluble tumor necrosis factor receptor sTNF RII.  Ref: 12708230 Int J Hyg Environ Health, 2003
- Increased soluble interleukin 6  [ **IL6** ]  receptor  [ **IL6R** ]  in vitreous fluid of proliferative vitreoretinopathy.  Ref: 12789531 Curr Eye Res, 2003
- Since hydroxamic acid based metalloprotease inhibitors prevent shedding of both TNFR1 and the interleukin 6  [ **IL6** ]  receptor  [ **IL6R** ]  IL 6Ra, we hypothesized that ARTS 1 might also regulate shedding of IL 6Ra, a member of the type I cytokine receptor superfamily that is structurally different from TNFR1.  Ref: 12748171 J Biol Chem< MedlineTA>J Biol Chem, 2003
- We have determined the percentage of alphabeta and gammadelta T cells by flow cytometry as well as serum interleukin 6  [ **IL6** ]  IL 6  [ **IL6** ]  and soluble interleukin 6  [ **IL6** ]  receptor  [ **IL6R** ]  sIL 6R levels by enzyme linked immunosorbent assay in kidney allograft recipients with acute, chronic or stable graft evolution.  Ref: 12828564 Scand J Immunol< MedlineTA>Scand J Immunol, 2003
